# Multiple brain metastases: resection with IORT versus adjuvant radiotherapy and concurrent irradiation of unresected lesions

**DOI:** 10.1186/s13014-025-02736-2

**Published:** 2025-10-21

**Authors:** Gero Wieger, Àlex Godó Jiménez, Stefanie Brehmer, Nima Etminan, Florian Stieler, Frank A. Giordano, Arne Mathias Ruder

**Affiliations:** 1 Department of Radiation Oncology, University Medical Centre Mannheim, Theodor-Kutzer-Ufer 1-3, 68167 Mannheim, Germany; 2 Department of Neurosurgery, University Medical Centre Mannheim, Theodor-Kutzer-Ufer 1-3, 68167 Mannheim, Germany; 3 DKFZ-Hector Cancer Institute, University Medical Centre Mannheim, Mannheim, Germany; 4 Mannheim Institute for Intelligent Systems in Medicine (MIISM), Medical Faculty Mannheim, Heidelberg University, Mannheim, Germany

**Keywords:** Brain metastases, Intraoperative radiotherapy (IORT), Stereotactic radiosurgery (SRS), Fractionated stereotactic radiotherapy (FSRT), Gamma Knife

## Abstract

**Background:**

Treatment of multiple brain metastases (BM) often involves surgical resection of one lesion combined with stereotactic radiotherapy (SRT) to the resection cavity and unresected BM. Intraoperative radiotherapy (IORT) is an emerging alternative for treating the resection cavity, potentially dosimetric benefits. This study aimed to compare the dosimetry of treating the resection cavity with IORT versus adjuvant SRT when combined with SRT for additional unresected BM.

**Methods:**

Ten patients with BM who received adjuvant SRT to the resection cavity plus concurrent SRT to additional BM (adjuvant SRT + SRT to BM group) and 4 patients with IORT and SRT to BM were identified. Post-hoc IORT plans were calculated for IORT patients and summed with corresponding SRT plans. Additionally, patients from the adjuvant SRT + SRT to BM group served as templates for IORT simulation and the simulated IORT plans were also summed with the corresponding plans for SRT to residual BM. The simulated IORT cases were then pooled with the actual IORT cases, forming the IORT + SRT to BM group. Brain dose exposure (V12Gy, V18Gy, V20Gy, V24Gy, V25Gy, and V30Gy) and maximum doses (D0.035 cm³) to the brainstem and optic tract were evaluated.

**Results:**

The IORT + SRT to BM group showed significantly lower brain dose exposure across all metrics (V12Gy-V30Gy) compared to the adjuvant SRT + SRT to BM group (*p* < 0.05 for all). Maximum doses to organs at risk were lower in the IORT + SRT to BM group, with mean D0.035 cm³ reduced by 37.7% for the brainstem (3.51 Gy vs. 5.65 Gy, *p* = 0.292) and significantly reduced by 57.5% for the optic tract (0.89 Gy vs. 2.10 Gy, *p* = 0.040).

**Conclusions:**

IORT in combination with SRT for additional BM demonstrated superior dosimetric properties compared to adjuvant cavity SRT plus concurrent SRT to BM.

**Trial registration:**

Not applicable (Retrospective dosimetric study).

## Introduction

Brain metastases (BM) present a considerable challenge in the field of neuro-oncology, with up to 40% of patients developing BM over the course of their disease [1]. Advances in diagnostic imaging and the advent of novel systemic therapies have led to prolonged overall survival (OS), but have also contributed to an increased incidence of brain metastases [2, 3]. Notably, the presence of BM does not necessarily reduce OS [4].

Nevertheless, ensuring effective local tumor control remains pivotal to preserving neurological function and quality of life [5]. Local radiotherapy has gained considerable support as an adjuvant to surgical resection, given that recurrence rates can approach 50% when no postoperative radiation is administered, even after complete tumor removal [6–8]. Standard management thus consists of surgical resection followed by stereotactic radiotherapy (SRT), for example as stereotactic radiosurgery (SRS) or fractionated stereotactic radiation therapy (FSRT), approaches that allow for high-dose irradiation to the resection cavity while sparing neighboring healthy tissue [9].

Intraoperative radiotherapy (IORT) has recently emerged as a potential alternative for treating resection cavities, with prospective and retrospective data indicating promising rates of local control alongside a favorable safety profile [10–13]. The highly targeted nature of IORT offers distinct dosimetric advantages, delivering high radiation doses directly to the resection cavity while sparing adjacent healthy brain tissue — even in comparison to highly conformal SRS [14, 15]. In clinical practice, patients undergoing resection for BM frequently present with additional, non-resectable metastases diagnosed simultaneously. In such cases, treatment often involves adjuvant SRT to the resection cavity in combination with SRT to the residual BM.

This study aims to assess the dosimetric differences between two strategies for the treatment of a resection cavity and simultaneously present BM in situ: adjuvant SRT to the resection cavity versus IORT, both combined with SRT to the unresected lesions.

## Materials and methods

### Patient selection

We included 14 patients who underwent BM resection between 2017 and 2024 with at least one additional BM in the magnetic resonance imaging (MRI) obtained before resection, and a subsequent indication for SRT to the additional BM. The patients received either adjuvant Gamma Knife SRT to the resection cavity (*n* = 10) or IORT (*n* = 4). Patients who did not receive SRT for the additional BM, underwent cerebellar resection, had multiple resection cavities, or had incomplete data were excluded from the analysis.

### SRT to the resection cavity

Patients receiving adjuvant SRT were treated using the Leksell Gamma Knife Icon (Elekta AB, Stockholm, Sweden) with either frame fixation (FF) or thermoplastic mask fixation (MF). A dedicated radiotherapy planning MRI, together with a planning CT, was acquired for every patient. For FF, the planning target volume (PTV) encompassed the resection cavity with a 2 mm margin and any adjacent T1-contrast-enhancing lesion. Conversely, for MF, an additional 1 mm margin was added to these structures to define the PTV. Treatment plans were then optimized for conformity and target coverage using Leksell GammaPlan (Elekta AB, Stockholm, Sweden). The dose was prescribed to the 50–80% isodose. For cases receiving 30 Gy in 3 fractions, a staged SRT approach was applied, with an interval of 14 days between fractions to allow for volumetric reduction and re-planning prior to subsequent fractions.

### IORT to the resection cavity

For patients treated with IORT, a dose of 30 Gy was applied with a spherical applicator using the INTRABEAM system (Zeiss AG, Oberkochen, Germany). Dose was prescribed to the applicator surface and safety margins of 1.5 cm to any organs at risk (OAR; brainstem, optic chiasm or optic nerves) were confirmed using a neuronavigation system (Brainlab, Munich, Germany) before IORT administration.

### SRT to additional brain metastases

After radiotherapy planning MRI and CT, all patients were treated using the Leksell Gamma Knife Icon with either FF or MF. For FF, the PTV encompassed all T1-contrast-enhancing lesions and, for MF, an additional 1 mm margin was added to these structures to define the PTV. Treatment plans were optimized for conformity and target coverage using Leksell GammaPlan. Dose was prescribed to the 50–80% isodose.

### IORT treatment reconstruction, IORT simulation and dose summation

Post-hoc IORT treatment plans for patients who had received IORT were generated using Radiance (GMV SA, Madrid, Spain). A spherical applicator with the same diameter used during the actual IORT was simulated and placed inside the resection cavity delineated on the planning MRI.

**Fig. 1:**
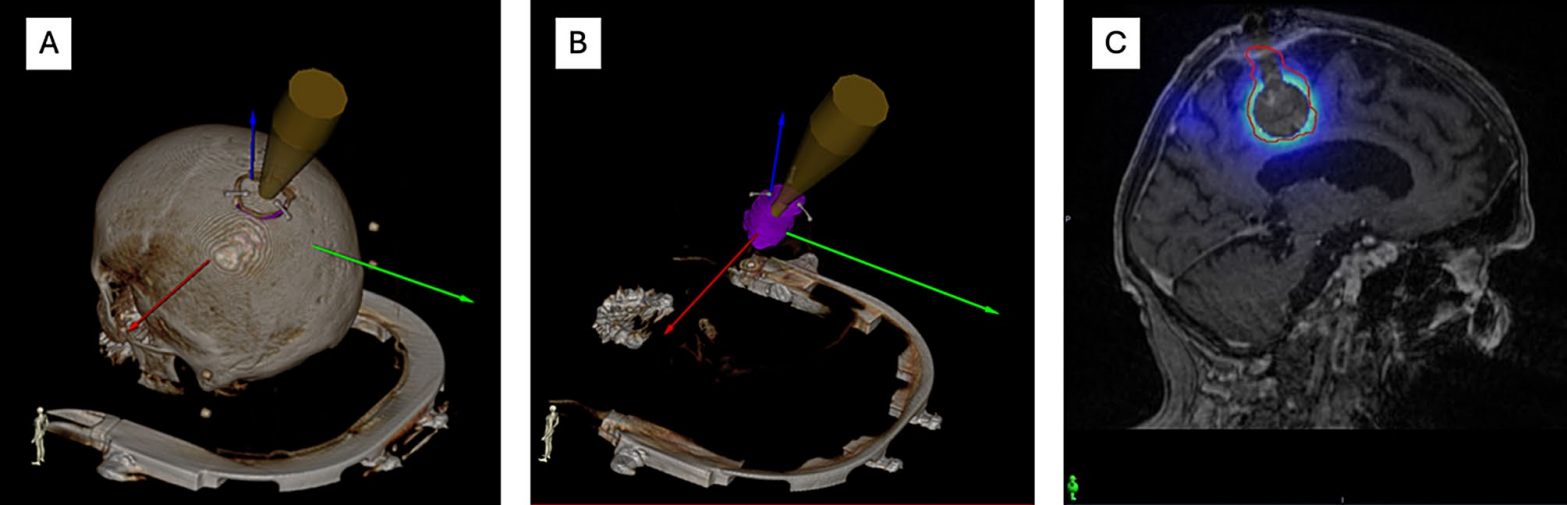
Simulated intraoperative radiotherapy (IORT) planning for a resection cavity. (**A**) Applicator alignment along the surgical path with skull visible. (**B**) Applicator placement within the resection cavity (pink). (**C**) Resulting dose distribution calculated in Velocity planning software

For patients who had received adjuvant SRT, an IORT treatment was simulated in Radiance with an applicator size chosen to fit the resection cavity delineated in the planning MRI. Dose distribution was calculated via Monte Carlo algorithm with the planning CT co-registered to the MRI. An example is depicted in Fig. 1.

**Fig. 2:**
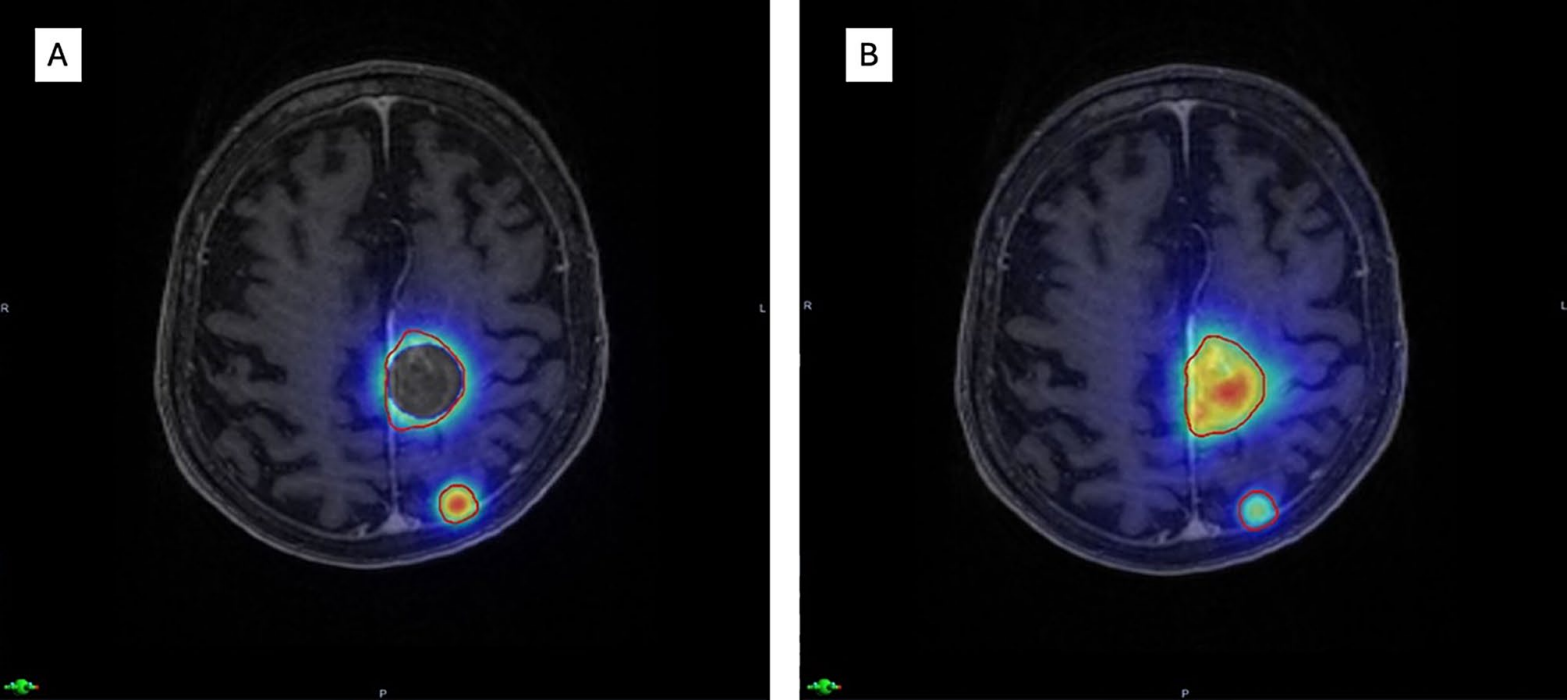
Example cumulative dose distributions for combined treatments. (**A**) Simulated IORT (30 Gy) to the parasagittal left resection cavity combined with SRT (22 Gy/1 fx) to the left parietal metastasis. Heat map normalized to a maximum dose of 44 Gy. (**B**) Adjuvant SRT (30 Gy/3 fx) to the resection cavity combined with SRT (22 Gy/1 fx) to the metastasis. Heat map normalized to a maximum dose of 60 Gy

Then, summations of reconstructed or simulated IORT plans and the plans of SRT to additional BM were performed after rigid co-registration of all planning images in Velocity (Varian, Palo Alto, USA). An exemplary sum plan is depicted in Fig. 2.

Subsequently, patients treated with actual IORT and simulated IORT cases were pooled to form the IORT + SRT to BM group (*n* = 14) that was compared to the adjuvant SRT + SRT to BM group (*n* = 10).

### Statistical analysis

All statistical analyses were conducted using R version 2024.12.1 + 563 (R Foundation for Statistical Computing, Vienna, Austria). The following R packages were used: tidyverse (including dplyr, tidyr, and ggplot2) for data processing and visualization, readxl for data import, ggsignif for significance annotation in plots, patchwork for plot composition, and kableExtra for advanced table formatting. Continuous variables are reported as means with standard deviations (SD) or medians with interquartile ranges (IQR), as appropriate. Between-group comparisons were performed using Welch’s t-test or the Wilcoxon rank-sum test, depending on the results of the Shapiro-Wilk test for normality. Effect sizes are presented as Cohen’s d (for parametric comparisons) or Cliff’s delta (δ) (for nonparametric comparisons). A significance threshold of *p* ≤ 0.05 was applied.

### Dosimetric parameters and endpoints

The brain, brainstem and optic tract comprising the optic chiasm and both optic nerves were delineated in the radiotherapy planning MRI using Velocity. The sum doses were analyzed in Velocity to assess the exposure of the critical structures. The primary areas of interest encompass the disparities in brain V12Gy, V18Gy, V20Gy, V24Gy, V25Gy, V30Gy, between cavity SRT and IORT plus BM directed SRT plans. Brain was defined as healthy brain tissue including the target volume, as healthy parenchyma is inherently part of the target volume definition in adjuvant cavity SRT. Additionally, maximum dose (D0.035 cm³) and mean dose (Dmean) were assessed for the brainstem and optic tract.

### Ethics

Local institutional review board approval (Ethikkommission II, Mannheim Medical Faculty, Heidelberg University) was obtained before data collection. Prior to analysis, all datasets were anonymized, and this manuscript contains no personally identifiable information, in accordance with the principles of the Declaration of Helsinki.

## Results

### Patient characteristics and treatment parameters

Our cohort comprised of 10 patients with adjuvant cavity SRT and 4 patients treated with IORT, all in combination with SRT to residual BM. The 10 patients with adjuvant SRT to the resection cavity served as templates for IORT simulations and were pooled with 4 patients treated with actual IORT. The simulated IORT cases did not differ significantly from the actual IORT patients for dose exposure to the brain or brainstem maximum dose, while maximum dose to the optic tract was lower for the patients with actual IORT (mean: 0.20 Gy) compared to simulated IORT cases (mean: 0.71), with a significant difference (Wilcoxon rank-sum test, *p* = 0.04). Details of the comparison are listed in the Appendix Table 1. Patient details and treatment parameters are summarized in Table 1.

**Table 1:** Patient characteristics and treatment parameters

Characteristics	Value	Median	Min	Max	SD
**Demographics**					
Overall Cohort	*n* = 14				
**Sex**					
Female	*n* = 8				
Male	*n* = 6				
**Primary Tumor Type**					
Non-small cell lung cancer	*n* = 12				
Mesothelioma	*n* = 1				
Renal cell carcinoma	*n* = 1				
**Treatment Parameters**					
Age at Surgery (years)		70	54	83	8.8
Time from Surgery to SRT Start (days)		21	1	56	13.1
Number of Metastases Treated		1	1	3	0.6
Cavity Volume (cm³)		9.86	3.71	19.90	5.85
Dose per Fraction (Gy)		10	5	10	2.6
True applicator diameter (mm)		20	20	30	5.0
Simulated applicator diameter (mm)		20	15	30	5.3

Baseline patient demographics, tumor characteristics, and treatment parameters for the Adjuvant SRT + SRT to BM group (*n* = 10) and the combined IORT + SRT to BM group (*n* = 14). Values presented as n for categorical variables or median (minimum - maximum) ± Standard Deviation (SD) for continuous variables, as appropriate

### Comparisons of dosimetric parameters between treatment groups

**Fig. 3:**
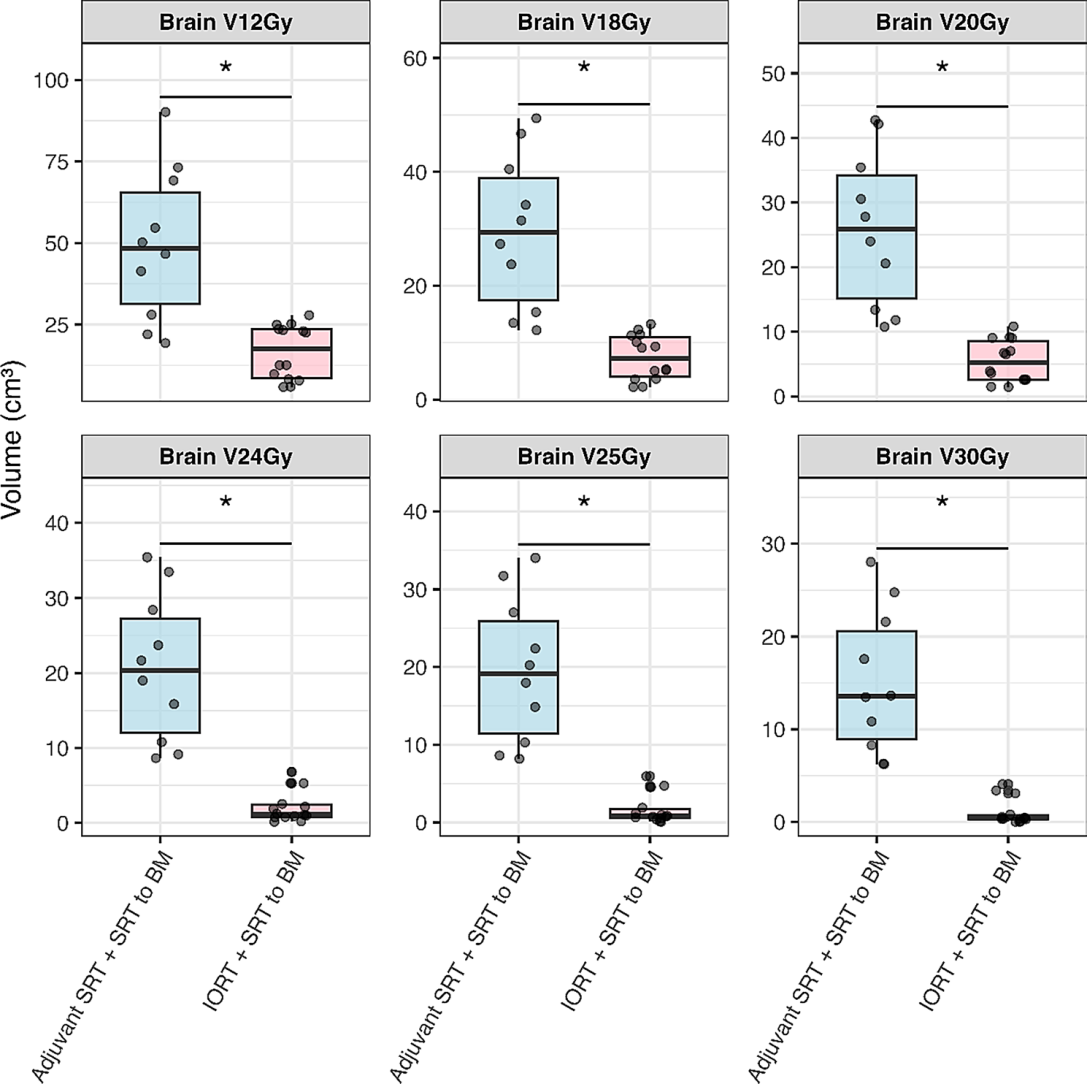
Brain dose exposure comparison between treatment groups. Brain volume (cm³) receiving specified doses (V12Gy – V30Gy). Comparison between Adjuvant SRT + SRT to BM (*n* = 10) and IORT + SRT to BM (*n* = 14). Boxplots show median (line), interquartile range (box), whiskers (extending to 1.5x IQR), and outliers (dots). Asterisks (*) indicate statistically significant differences (*p* ≤ 0.05)

In patients who received adjuvant SRT + SRT to BM, dose exposure to the brain was significantly higher than in patients treated or simulated with IORT + SRT to BM. Dosimetric parameters of the groups are listed in Table 2. All patients treated or simulated with IORT + SRT to BM achieved V20Gy exposures < 20 cc, while 7 of 10 patients who received adjuvant SRT + SRT to BM showed V20Gy exposures > 20 cc. The dosimetric comparisons between the groups are depicted in Fig. 3. Details of the statistical analysis can be found in the Appendix Table 2.

**Fig. 4:**
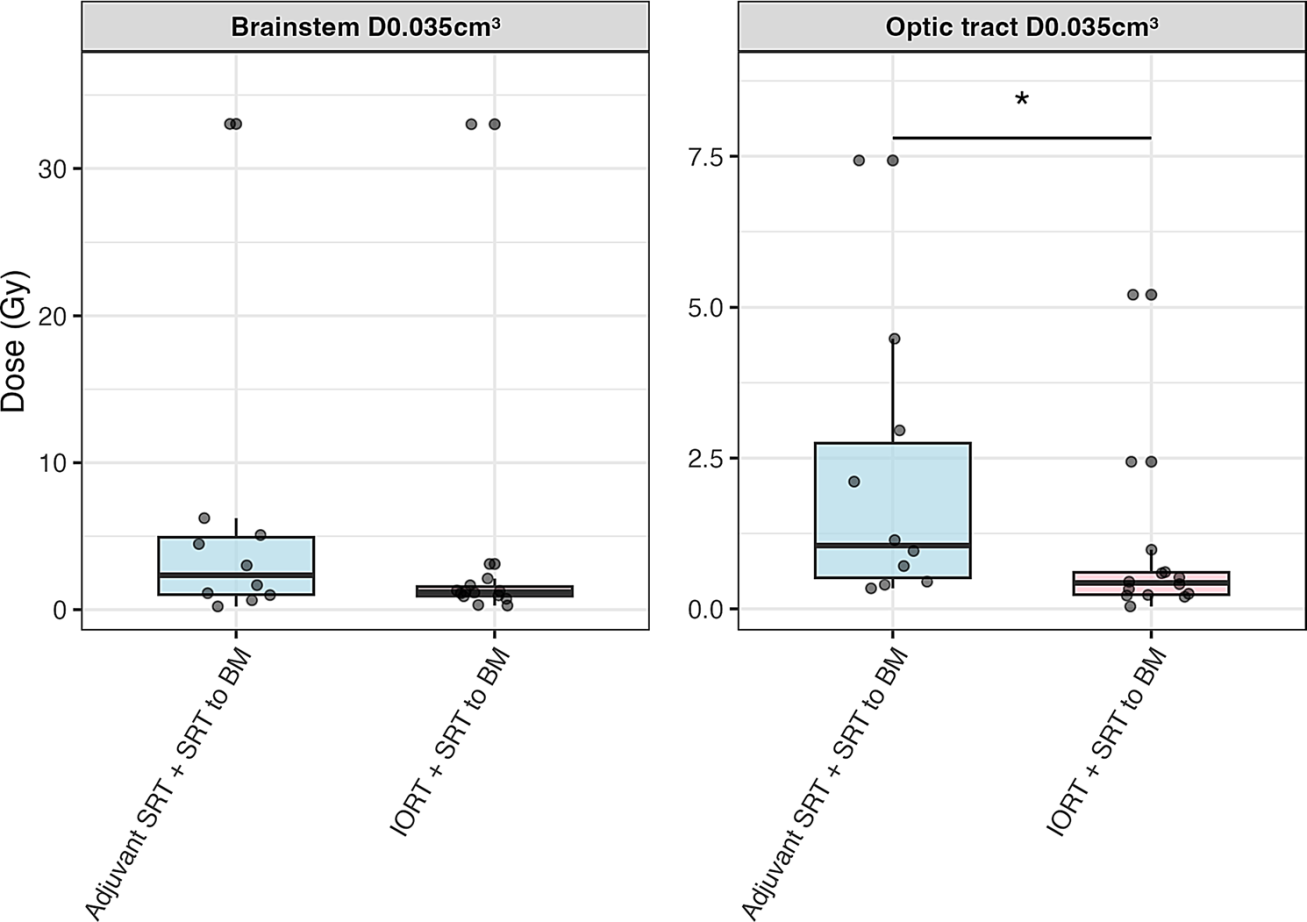
Organ at risk maximum dose comparison between treatment groups. Maximum dose (D0.035 cm³) for brainstem and optic tract. Comparison between Adjuvant SRT + SRT to BM (*n* = 10) and IORT + SRT to BM (*n* = 14). Boxplots show median (line), interquartile range (box), whiskers (extending to 1.5x IQR), and outliers (dots). Asterisks (*) indicate statistically significant differences (*p* ≤ 0.05)

Maximum dose to optic tract was significantly higher for patients who received adjuvant SRT + SRT to BM compared to the IORT group. The comparison is shown in Fig. 4.

**Table 2:** Comparison of dosimetric parameters between treatment groups

Variable	Adjuvant SRT + SRT to BM (*n* = 10)	IORT + SRT to BM (*n* = 14)
Brain V12Gy (cm³)	49.48 ± 23.17	16.67 ± 8.30
Brain V18Gy (cm³)	29.43 ± 13.47	7.42 ± 3.93
Brain V20Gy (cm³)	25.91 ± 11.93	5.47 ± 3.23
Brain V24Gy (cm³)	20.62 ± 9.74	2.15 ± 2.12
Brain V25Gy (cm³)	19.53 ± 9.32	1.67 ± 1.92
Brain V30Gy (cm³)	15.07 ± 7.72	1.00 ± 1.39
Brainstem D0.035 cm³ (Gy)	5.65 ± 9.84	3.51 ± 8.52
Optic tract D0.035 cm³ (Gy)	2.10 ± 2.30	0.89 ± 1.38

Comparison of dosimetric parameters (Brain V12Gy-V30Gy [cm³], brainstem D0.035 cm³ [Gy], optic tract D0.035 cm³ [Gy]) between the Adjuvant SRT + SRT to BM group (*n* = 10) and the combined IORT + SRT to BM group (*n* = 14). Values reported as Mean ± Standard Deviation

## Discussion

This study provides the first dosimetric comparison of IORT versus SRT to the resection cavity combined with SRT for residual BM. The rationale for this comparison lies in the potential clinical advantages of IORT in the setting of multiple BM with indication for resection of at least one BM. IORT to BM has been associated with lower rates of radiation necrosis — reported as low as 2.9% — while achieving local control rates comparable to conventional stereotactic techniques [11–13, 15, 16]. In clinical scenarios where SRT is required for additional BM alongside resection and IORT, dosimetric advantages become increasingly relevant. In the context of glioblastoma treatment, a recent dosimetric analysis directly compared an additive pre-operative SRT boost with additive IORT in combination with standard external beam radiotherapy. The study demonstrated that IORT offers a superior dose distribution, primarily due to its steep dose fall-off, which significantly spares adjacent healthy tissue [17]. IORT is inherently independent of post-surgical resection cavity variability, thereby avoiding unnecessarily large target volumes in the adjuvant setting and thus further sparing healthy tissue [18].

Our analysis, based on the first real-world IORT cohort supplemented by additional simulated cases, demonstrated a significant reduction in brain dose exposure compared to patients receiving Adjuvant SRT + SRT to BM. Given that IORT and SRT for residual BM were delivered at different time points and that SRT included fractionated treatment, dose-volume parameters established for fractionated SRT, such as V18Gy, V20Gy, V24Gy, and V30Gy, appear most appropriate for estimating radionecrosis risk [19–22]. IORT in place of cavity SRT resulted in significant reductions of V18Gy, V20Gy, V24Gy, and V30Gy. Notably, none of the real or simulated IORT cases exceeded the brain dose thresholds to prevent radionecrosis established in the literature. Additionally, with contemporary systemic therapies extending survival in patients with BM, neurocognitive function has become an increasingly critical consideration. A global reduction in brain dose exposure is expected to help preserve patients’ neurocognitive function [23–25].

With regard to organs at risk (OAR), IORT resulted in a reduction in average maximal dose exposure by 37.7% for the brainstem (3.51 Gy vs. 5.65 Gy ) and by 57.5% for the optic tract (0.89 Gy vs. 2.10 Gy), with the latter showing statistical significance. Even though dose tolerance limits of chiasm an optical nerves were not reached, this highlights the potential of IORT combined with SRT for residual metastases to offer greater dosimetric flexibility, particularly in cases where BM are located in close proximity to critical OAR.

The study design, which includes both actual patients treated with IORT or adjuvant SRT + SRT to BM as well as simulated IORT cases, presents certain limitations that should be acknowledged when interpreting these findings. However, the similarity of results between real IORT patients and simulated IORT cases supports the validity as a comparison cohort to cavity SRT patients. As with all dosimetric post hoc analyses, it should be noted that, despite meticulous dose-matching using advanced treatment planning software, inherent uncertainties in simulation and dose calculation remain. Prospective randomized data are expected from the LEXIMATE trial, which also includes IORT combined with SRT to concurrent brain metastases [26].

## Conclusion

IORT combined with SRT to residual BM demonstrated superior dosimetric properties compared to adjuvant cavity SRT, with significantly reduced dose exposure to the healthy brain and optic tract. These findings support further investigation of IORT as a treatment strategy in patients with multiple brain metastases.

## Appendix

**Appendix Table 1:** Simulated IORT + SRT to BM vs. Actual IORT + SRT to BM

Variable	Test	*p*-value	Effect Size	Sig
Brain V12Gy (cm³)	Wilcoxon	0.945	δ = -0.05	
Brain V18Gy (cm³)	Wilcoxon	0.839	δ = 0.10	
Brain V20Gy (cm³)	Wilcoxon	0.839	δ = 0.10	
Brain V24Gy (cm³)	Wilcoxon	0.454	δ = 0.30	
Brain V25Gy (cm³)	Wilcoxon	0.635	δ = 0.20	
Brain V30Gy (cm³)	Wilcoxon	1.000	δ = 0.00	
Brainstem D0.035 cm³ (Gy)	Wilcoxon	0.240	δ = 0.45	
Optic tract D0.035 cm³ (Gy)	Wilcoxon	0.024	δ = 0.80	*

Statistical details comparing dosimetric parameters (brain V12Gy-V30Gy, brainstem D0.035 cm³, optic tract D0.035 cm³) for patients actually treated with IORT plus SRT (*n* = 4) versus simulated IORT cases derived from the adjuvant SRT cohort (*n* = 10). Includes statistical test performed (e.g., Wilcoxon rank-sum), p-values, and effect sizes (e.g., Cliff’s delta)

**Appendix Table 2:** Statistical Comparison Between Combined IORT and Adjuvant SRT Groups

Variable	Test	*p*-value	Effect Size	Sig
Brain V12Gy (cm³)	Wilcoxon	0.001	δ = 0.80	*
Brain V18Gy (cm³)	Welch t-test	< 0.001	d = 2.33	*
Brain V20Gy (cm³)	Welch t-test	< 0.001	d = 2.46	*
Brain V24Gy (cm³)	Wilcoxon	< 0.001	δ = 1.00	*
Brain V25Gy (cm³)	Wilcoxon	< 0.001	δ = 1.00	*
Brain V30Gy (cm³)	Wilcoxon	< 0.001	δ = 1.00	*
Brainstem D0.035 cm³ (Gy)	Wilcoxon	0.292	δ = 0.26	
Optic tract D0.035 cm³ (Gy)	Wilcoxon	0.040	δ = 0.51	*

Detailed statistical results for the comparison of dosimetric parameters (brain V12Gy-V30Gy, brainstem D0.035 cm³, optic tract D0.035 cm³) between the combined IORT + SRT group (*n* = 14) and the Adjuvant SRT + SRT to BM group (*n* = 10). Includes statistical test performed (e.g., Welch’s t-test, Wilcoxon rank-sum), p-values, and effect sizes (e.g., Cohen’s d, Cliff’s delta). In two Adjuvant SRT + SRT to BM cases V30Gy was 0 cm³ while Dmax was 27.17 Gy and 27.39 Gy, respectively

**Appendix Table 3:** Per-patient prescription doses for cavity treatment and concurrent irradiation of unresected brain metastases

Patient	Cavity SRT Dose	IORT Dose	BM 1 Dose	BM 2 Dose	BM 3 Dose
1	10 Gy × 3*		10 Gy × 3*	10 Gy × 3*	
2	10 Gy × 3*		22 Gy × 1	22 Gy × 1	10 Gy × 3*
3	10 Gy × 3*		22 Gy × 1		
4	10 Gy × 3*		22 Gy × 1		
5	10 Gy × 3*		10 Gy × 3*	22 Gy × 1	
6	10 Gy × 3*		22 Gy × 1		
7	5 Gy × 6°		20 Gy × 1		
8	5 Gy × 6°		22 Gy × 1		
9	5 Gy × 7°		22 Gy × 1		
10	5 Gy × 7°		9 Gy × 3°	9 Gy × 3°	
11		30 Gy × 1	22 Gy × 1	22 Gy × 1	
12		30 Gy × 1	22 Gy × 1		
13		30 Gy × 1	22 Gy × 1		
14		30 Gy × 1	22 Gy × 1		

Doses are reported as Gy × fractions (Gy × fx) for the resection cavity (adjuvant SRT or IORT) and up to three unresected metastases (BM 1–3). * Staged FSRT: 30 Gy in 3 fx with 14-day intervals (q14d) and re-planning before each fraction. ° FSRT on consecutive days. IORT doses were prescribed to the applicator surface (INTRABEAM system). Empty cells indicate cases not treated or not applicable. Values reflect real-world prescriptions (no post-hoc re-planning or dose normalization)

## Data Availability

The datasets used and/or analysed during the current study (primarily anonymised dosimetric data) are available from the corresponding author (GW) on reasonable request.

## References

[1] Cagney DN, Martin AM, Catalano PJ, Redig AJ, Lin NU, Lee EQ, et al. Incidence and prognosis of patients with brain metastases at diagnosis of systemic malignancy: a population-based study. Neuro Oncol. 2017;19(11):1511–21. 28444227 10.1093/neuonc/nox077 PMC5737512

[2] Antonia SJ, Villegas A, Daniel D, Vicente D, Murakami S, Hui R, et al. Overall survival with durvalumab after chemoradiotherapy in stage III NSCLC. N Engl J Med. 2018;379(24):2342–50. 30280658 10.1056/NEJMoa1809697

[3] Nayak L, Lee EQ, Wen PY. Epidemiology of brain metastases. Curr Oncol Rep. 2012;14(1):48–54. 22012633 10.1007/s11912-011-0203-y

[4] Yamamoto M, Sato Y, Serizawa T, Kawabe T, Higuchi Y, Nagano O, et al. Subclassification of recursive partitioning analysis Class II patients with brain metastases treated radiosurgically. Int J Radiat Oncol Biol Phys. 2012;83(5):1399–405. 22209155 10.1016/j.ijrobp.2011.10.018

[5] Verhaak E, Gehring K, Hanssens PEJ, Sitskoorn MM. Health-related quality of life of patients with brain metastases selected for stereotactic radiosurgery. J Neurooncol. 2019;143(3):537–46. 31073966 10.1007/s11060-019-03186-z PMC6591192

[6] Brown PD, Ballman KV, Cerhan JH, Anderson SK, Carrero XW, Whitton AC, et al. Postoperative stereotactic radiosurgery compared with whole brain radiotherapy for resected metastatic brain disease (NCCTG N107C/CEC·3): a multicentre, randomised, controlled, phase 3 trial. Lancet Oncol. 2017;18(8):1049–60. 28687377 10.1016/S1470-2045(17)30441-2 PMC5568757

[7] Combs SE, Bilger A, Diehl C, Bretzinger E, Lorenz H, Oehlke O, et al. Multicenter analysis of stereotactic radiotherapy of the resection cavity in patients with brain metastases. Cancer Med. 2018;7(6):2319–27. 29696815 10.1002/cam4.1477 PMC6010760

[8] Kocher M, Soffietti R, Abacioglu U, Villa S, Fauchon F, Baumert BG, et al. Adjuvant whole-brain radiotherapy versus observation after radiosurgery or surgical resection of one to three cerebral metastases: results of the EORTC 22952–26001 study. J Clin Oncol. 2011;29(2):134–41. 21041710 10.1200/JCO.2010.30.1655 PMC3058272

[9] Mahajan A, Ahmed S, McAleer MF, Weinberg JS, Li J, Brown P, et al. Post-operative stereotactic radiosurgery versus observation for completely resected brain metastases: a single-centre, randomised, controlled, phase 3 trial. Lancet Oncol. 2017;18(8):1040–8. 28687375 10.1016/S1470-2045(17)30414-X PMC5560102

[10] de Castro DG, Sanematsu PI Jr., Pellizzon ACA, Suzuki SH, Fogaroli RC, Dias JES Jr., et al. Intraoperative radiotherapy for brain metastases: first-stage results of a single-arm, open-label, phase 2 trial. J Neurooncol. 2023;162(1):211–5. 36826700 10.1007/s11060-023-04266-x

[11] Layer JP, Hamed M, Potthoff AL, Dejonckheere CS, Layer K, Sarria GR, et al. Outcome assessment of intraoperative radiotherapy for brain metastases: results of a prospective observational study with comparative matched-pair analysis. J Neurooncol. 2023;164(1):107–16. 37477822 10.1007/s11060-023-04380-w PMC10462513

[12] Layer JP, Shiban E, Brehmer S, Diehl CD, de Castro DG, Hamed M, et al. Multicentric assessment of safety and efficacy of combinatorial adjuvant brain metastasis treatment by intraoperative radiation therapy and immunotherapy. Int J Radiat Oncol Biol Phys. 2024;118(5):1552–62. 38199383 10.1016/j.ijrobp.2024.01.009

[13] Brehmer S, Sarria GR, Würfel S, Sulejmani A, Schneider F, Clausen S, et al. INTRAMET: results of a prospective, single-arm, open-label phase II trial of intraoperative radiotherapy after resection of brain metastases. J Clin Oncol. 2023;41(16suppl):2031.

[14] Vargo JA, Sparks KM, Singh R, Jacobson GM, Hack JD, Cifarelli CP. Feasibility of dose escalation using intraoperative radiotherapy following resection of large brain metastases compared to post-operative stereotactic radiosurgery. J Neurooncol. 2018;140(2):413–20. 30094718 10.1007/s11060-018-2968-4 PMC6368183

[15] Cifarelli CP, Brehmer S, Vargo JA, Hack JD, Kahl KH, Sarria-Vargas G, Giordano FA. Intraoperative radiotherapy (IORT) for surgically resected brain metastases: outcome analysis of an international cooperative study. J Neurooncol. 2019;145(2):391–7. 31654248 10.1007/s11060-019-03309-6 PMC7007764

[16] Dejonckheere CS, Layer JP, Hamed M, Layer K, Glasmacher A, Friker LL, et al. Intraoperative or postoperative stereotactic radiotherapy for brain metastases: time to systemic treatment onset and other patient-relevant outcomes. J Neurooncol. 2023;164(3):683–91. 37812290 10.1007/s11060-023-04464-7 PMC10589145

[17] Sarria GR, Smalec Z, Muedder T, Holz JA, Scafa D, Koch D, et al. Dosimetric comparison of upfront boosting with stereotactic radiosurgery versus intraoperative radiotherapy for glioblastoma. Front Oncol. 2021;11:759873. 34778080 10.3389/fonc.2021.759873 PMC8581360

[18] Rogers CM, Jones PS, Weinberg JS. Intraoperative MRI for brain tumors. J Neurooncol. 2021;151(3):479–90. 33611714 10.1007/s11060-020-03667-6

[19] Milano MT, Grimm J, Niemierko A, Soltys SG, Moiseenko V, Redmond KJ, et al. Single- and multifraction stereotactic radiosurgery Dose/Volume tolerances of the brain. Int J Radiat Oncol Biol Phys. 2021;110(1):68–86. 32921513 10.1016/j.ijrobp.2020.08.013 PMC9387178

[20] Faruqi S, Ruschin M, Soliman H, Myrehaug S, Zeng KL, Husain Z, et al. Adverse radiation effect after hypofractionated stereotactic radiosurgery in 5 daily fractions for surgical cavities and intact brain metastases. Int J Radiat Oncol Biol Phys. 2020;106(4):772–9. 31928848 10.1016/j.ijrobp.2019.12.002

[21] Minniti G, Niyazi M, Andratschke N, Guckenberger M, Palmer JD, Shih HA, et al. Current status and recent advances in resection cavity irradiation of brain metastases. Radiat Oncol. 2021;16(1):73. 33858474 10.1186/s13014-021-01802-9 PMC8051036

[22] Gondi V, Bauman G, Bradfield L, Burri SH, Cabrera AR, Cunningham DA, et al. Radiation therapy for brain metastases: an ASTRO clinical practice guideline. Practical Radiation Oncol. 2022;12(4):265–82. 10.1016/j.prro.2022.02.003 35534352

[23] Brown PD, Ballman KV, Cerhan JH, Anderson SK, Carrero XW, Whitton AC, et al. Postoperative stereotactic radiosurgery compared with whole brain radiotherapy for resected metastatic brain disease (NCCTG N107C/CEC.3): a multicentre, randomised, controlled, phase 3 trial. Lancet Oncol. 2017;18(8):1049–60. 28687377 10.1016/S1470-2045(17)30441-2 PMC5568757

[24] Kępka L, Tyc-Szczepaniak D, Bujko K, Olszyna-Serementa M, Michalski W, Sprawka A, et al. Stereotactic radiotherapy of the tumor bed compared to whole brain radiotherapy after surgery of single brain metastasis: results from a randomized trial. Radiother Oncol. 2016;121(2):217–24. 27793446 10.1016/j.radonc.2016.10.005

[25] Brown PD, Jaeckle K, Ballman KV, Farace E, Cerhan JH, Anderson SK, et al. Effect of radiosurgery alone vs radiosurgery with whole brain radiation therapy on cognitive function in patients with 1 to 3 brain metastases: A randomized clinical trial. JAMA. 2016;316(4):401–9. 27458945 10.1001/jama.2016.9839 PMC5313044

[26] Diehl CD, Acker G, Brehmer S, Ganslandt O, Giordano FA, Hamed M, et al. RTID-05. study protocol: a multicenter, international randomized confirmatory trial: low-energy x-ray intraoperative radiation therapy (lex-iort) vs. adjuvant fractionated stereotactic radiotherapy (fsrt) in the management of brain metastases (leximate). Neurooncology. 2024;26(Supplement8):viii273–viii.

